# A translational rat model for ex vivo lung perfusion of pre-injured lungs after brain death

**DOI:** 10.1371/journal.pone.0260705

**Published:** 2021-12-02

**Authors:** Judith E. van Zanden, Henri G. D. Leuvenink, Erik A. M. Verschuuren, Michiel E. Erasmus, Maximilia C. Hottenrott

**Affiliations:** 1 Department of Surgery, University Medical Center Groningen, Groningen, The Netherlands; 2 Department of Pulmonary Diseases, University Medical Center Groningen, Groningen, The Netherlands; 3 Department of Cardiothoracic Surgery, University Medical Center Groningen, Groningen, The Netherlands; 4 Department of Surgery, University Hospital of Regensburg, Regensburg, Germany; Imperial College Healthcare NHS Trust, UNITED KINGDOM

## Abstract

The process of brain death (BD) detrimentally affects donor lung quality. *Ex vivo* lung perfusion (EVLP) is a technique originally designed to evaluate marginal donor lungs. Nowadays, its potential as a treatment platform to repair damaged donor lungs is increasingly studied in experimental models. Rat models for EVLP have been described in literature before, yet the pathophysiology of BD was not included in these protocols and prolonged perfusion over 3 hours without anti-inflammatory additives was not achieved. We aimed to establish a model for prolonged EVLP of rat lungs from brain-dead donors, to provide a reliable platform for future experimental studies. Rat lungs were randomly assigned to one of four experimental groups (n = 7/group): 1) healthy, directly procured lungs, 2) lungs procured from rats subjected to 3 hours of BD and 1 hour cold storage (CS), 3) healthy, directly procured lungs subjected to 6 hours EVLP and 4), lungs procured from rats subjected to 3 hours of BD, 1 hour CS and 6 hours EVLP. Lungs from brain-dead rats showed deteriorated ventilation parameters and augmented lung damage when compared to healthy controls, in accordance with the pathophysiology of BD. Subsequent *ex vivo* perfusion for 6 hours was achieved, both for lungs of healthy donor rats as for pre-injured donor lungs from brain-dead rats. The worsened quality of lungs from brain-dead donors was evident during EVLP as well, as corroborated by deteriorated ventilation performance, increased lactate production and augmented inflammatory status during EVLP. In conclusion, we established a stable model for prolonged EVLP of pre-injured lungs from brain-dead donor rats. In this report we describe tips and pitfalls in the establishment of the rat EVLP model, to enhance reproducibility by other researchers.

## Introduction

Brain-dead, multi-organ donors have the potential to save multiple lives of patients suffering from end-stage organ failure. However, the process of brain death (BD) is a major factor in the deterioration of graft quality, to which donor lungs seem particularly susceptible [1]. As a result, only 20–30% of the potential donor lungs meet the criteria to qualify for donation [2, 3]. In an attempt to narrow the global gap between donor lung supply and demand, the technique of *ex vivo* lung perfusion (EVLP) is increasingly applied to evaluate marginal donor lungs. Due to improved confidence in utilization of these lungs, experienced centers for clinical EVLP have reported an expansion of lung transplant activity by 70% [4]. Besides quality testing, EVLP is suggested to be a promising platform to treat donor lungs in an isolated manner, in an attempt to repair damaged donor lungs and possibly improve lung graft survival after transplantation [5]. Rat models for EVLP provide a valuable starting point for experimental research in the search for new applications and interventions of EVLP. Multiple rat EVLP models have previously been described in literature, yet the BD-induced pathophysiology and resulting lung damage was not included in these reports. In addition, EVLP of rat lungs for more than 3 hours without addition of anti-inflammatory additives has not been successfully established before. In this study, we aimed to develop a stable and reproducible rat EVLP model for prolonged perfusion of pre-injured donor lungs from brain-dead donors. This report details our successfully established protocol for BD and EVLP in rats, and describes tips and pitfalls to facilitate reproduction by other researchers.

## Materials and methods

### Rats, husbandry and care

Male inbred Lewis rats with a weight of 350–450 g were used, obtained from Harlan Laboratories, Melderslo, the Netherlands. Rats were housed under standard conditions, with *ad libitum* access to food and water. The environment was maintained at room temperature with a 12/12 light/dark cycle. This study was performed in compliance with the Principles of Laboratory Animal Care (NIH Publication No. 86–23, revised 1985) and the Dutch Law on Experimental Animal Care. The experiment was approved by the Institutional Animal Care and Use Committee of the University of Groningen (IACUC-RUG), approval No. 6826A. The protocol was designed to minimize animal suffering and accordingly, all operations were performed under general anesthesia.

### Experimental groups

**Fig 1:**
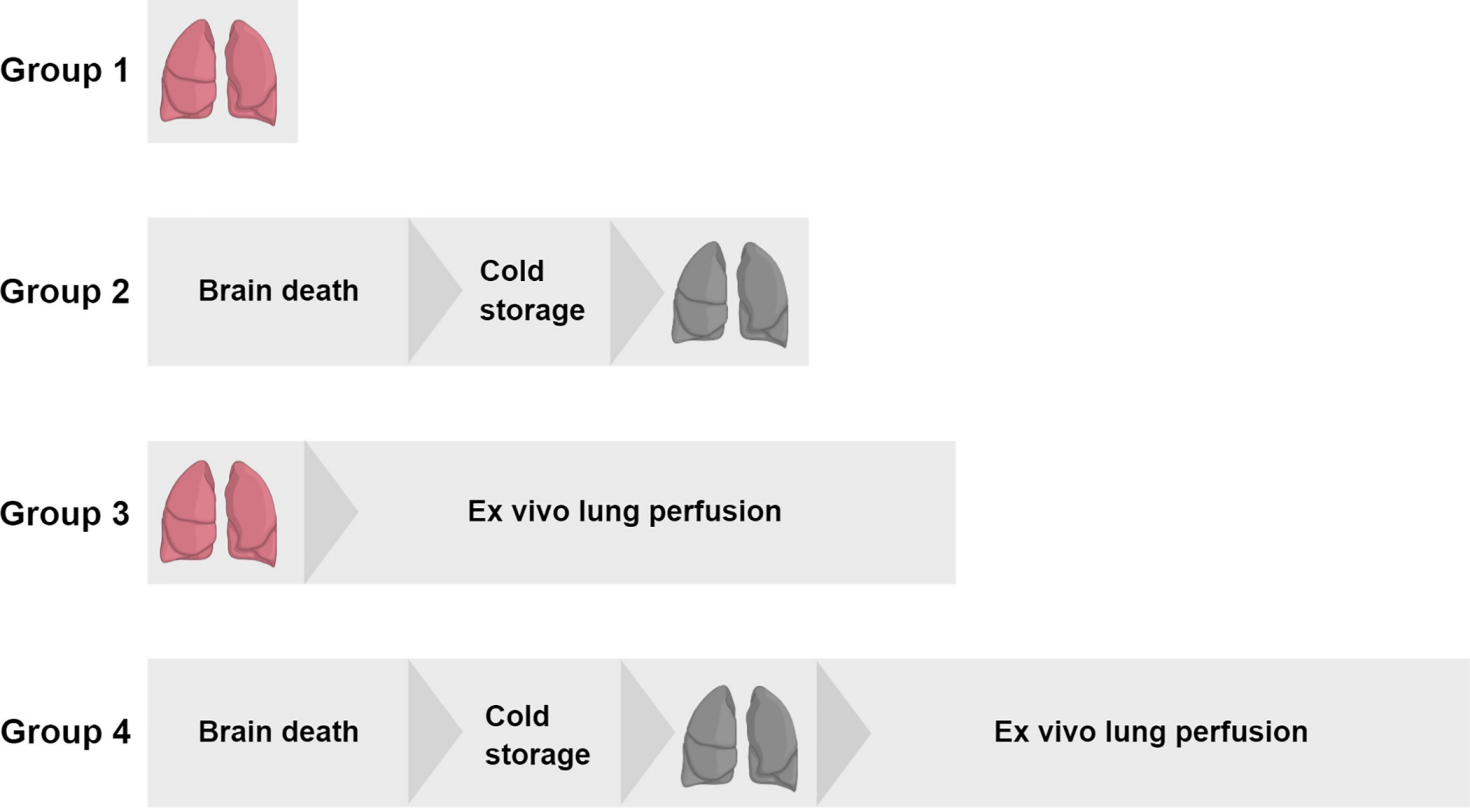
Experimental outline of the study. Lungs from donor rats were randomly assigned to one of four experimental groups (n = 7/group): 1) healthy, directly procured lungs, 2) lungs procured from rats subjected to 3 hours of brain death (BD) and 1 hour cold storage (CS), 3) healthy, directly procured lungs subjected to 6 hours *ex vivo* lung perfusion (EVLP) and 4), lungs procured from rats subjected to 3 hours of BD, 1 hour CS and 6 hours EVLP.

Since IL-6 is one of the most pronounced inflammatory markers in brain death, IL-6 expression was defined as the primary endpoint for power analyses [1]. Previous experiments by our group showed an absolute difference of 0.53 in lungs from brain-dead donor rats *versus* lungs from healthy donor rats and a variance of 0.3. With a pursued power of 0.9, 7 rats per group were required to reach statistical significance. Lungs from donor rats were randomly assigned to one of the following experimental groups (n = 7/group, Fig 1): 1) healthy, directly procured lungs, 2) lungs procured from rats subjected to 3 hours of BD and 1 hour cold storage (CS), 3) healthy, directly procured lungs subjected to 6 hours EVLP and 4), lungs procured from rats subjected to 3 hours of BD, 1 hour CS and 6 hours EVLP. A reduction of >15% of bodyweight prior to the experiment e.g. due to stress and changes in behavior, such as reduced exploratory activity, were defined as humane endpoints. Nevertheless, no rats required euthanasia at humane endpoints for this study.

### Brain death induction and lung procurement

The BD procedure was adapted from previously described models by our group [6, 7]. First, rats were anesthetized by subcutaneous administration of ketamine (75 mg/kg, Alfason B.V., Woerden, the Netherlands) and medetomidine hydrochloride (0.5 mg/kg, Orion Pharma, Mechelen, the Netherlands). For continuation of anesthesia, 1/4^th^ of the initial dose was administered every 15 min. Absence of muscle movement after toe pinch assessments confirmed the appropriate depth of anesthesia. The right femoral artery was cannulated for mean arterial pressure (MAP) measurements and the right femoral vein was cannulated for fluid administrations to maintain MAP >80 mmHg. In prone position, a craniotomy was performed a 4F Fogarty deflated balloon catheter (Edwards, Lifesciences, Irvine, USA) was inserted into the epidural space. Thereafter, the rat was turned to supine position for intubation and ventilation. A tracheotomy was performed and rats were intubated with a 14G polyethylene cannula. After connection of the ventilator (Babylog 8000 plus, Draeger, Lübeck, Germany) a recruitment maneuver was performed with positive inspiratory pressure (PIP) at 20 cmH_2_O and positive end-expiratory pressure (PEEP) at 15 cmH_2_O for 5 seconds. Thereafter, pressure-regulated volume controlled ventilation was initiated at the following settings: tidal volume (VT) of 7 ml/kg of bodyweight (BW), PEEP of 3 cmH_2_O, inspiratory/expiratory ratio (I:E) of 1:1 and fraction of inspired oxygen (FiO_2_) of 0.5. Rats were hyperventilated for 10 min on a respiratory rate of 150/min for preoxygenation prior to BD induction, and thereafter respiratory rate was reduced to a frequency of 133/min. BD was induced by manual inflation of the Fogarty catheter with 0.6 ml distilled water, continuously over 60 sec. The saline infusion pump was started at a rate of 3 ml/h to prevent hypovolemia due to ventilation and a heating pad established a body temperature of 37°C. At 15 min after BD induction, a second recruitment maneuver was performed as described above. Absence of corneal reflexes and toe pinch assessments were tested at 30 min after BD induction to confirm BD. Rats were stabilized for 3 hours at MAP >80 mmHg and in case of blood pressure drops, saline (Baxter B.V., Utrecht, the Netherlands) and Hydroxyethyl starch (HAES, Pharmacy-Fresenius Kabi, Bad Homburg, Germany) were manually administered at a maximum of 5 ml/hour saline and 2 ml/hour HAES. In group 1 and 3, the healthy control group, femoral vessel cannulation was omitted and lungs were immediately procured after intubation.

Before lung procurement, a recruitment maneuver was performed as described above. PEEP was lowered to 3 cmH_2_O and respiratory rate was lowered to 60/min to prevent lung damage during procurement. A median laparo-thoracotomy was performed and 1000 IU heparin (Leo Pharma B.V., Amsterdam, the Netherlands) were administered in the right ventricle. After incising the right ventricle, a flushed, air-bubble free cannula was inserted in the pulmonary artery and secured with the suture. The left ventricle was excised for drainage of lung flush with destruction of the mitral valve, to ensure outflow. Lungs were flushed with room temperature Perfadex (XVIVO Perfusion, Gothenburg, Sweden), at a pressure of 15 mmHg for 2 min. Subsequently, the heart-lung block was procured by dissecting the trachea from the larynx and separating the connective tissue between lungs and vertebrae. After a recruitment maneuver was performed as described above, PEEP was lowered to 5 cmH_2_O and the trachea was clamped with a bulldog. The inflated lungs were stored in a container with 100 ml cold Perfadex, placed on ice for 1 hour.

### Ex-vivo lung perfusion

**Fig 2:**
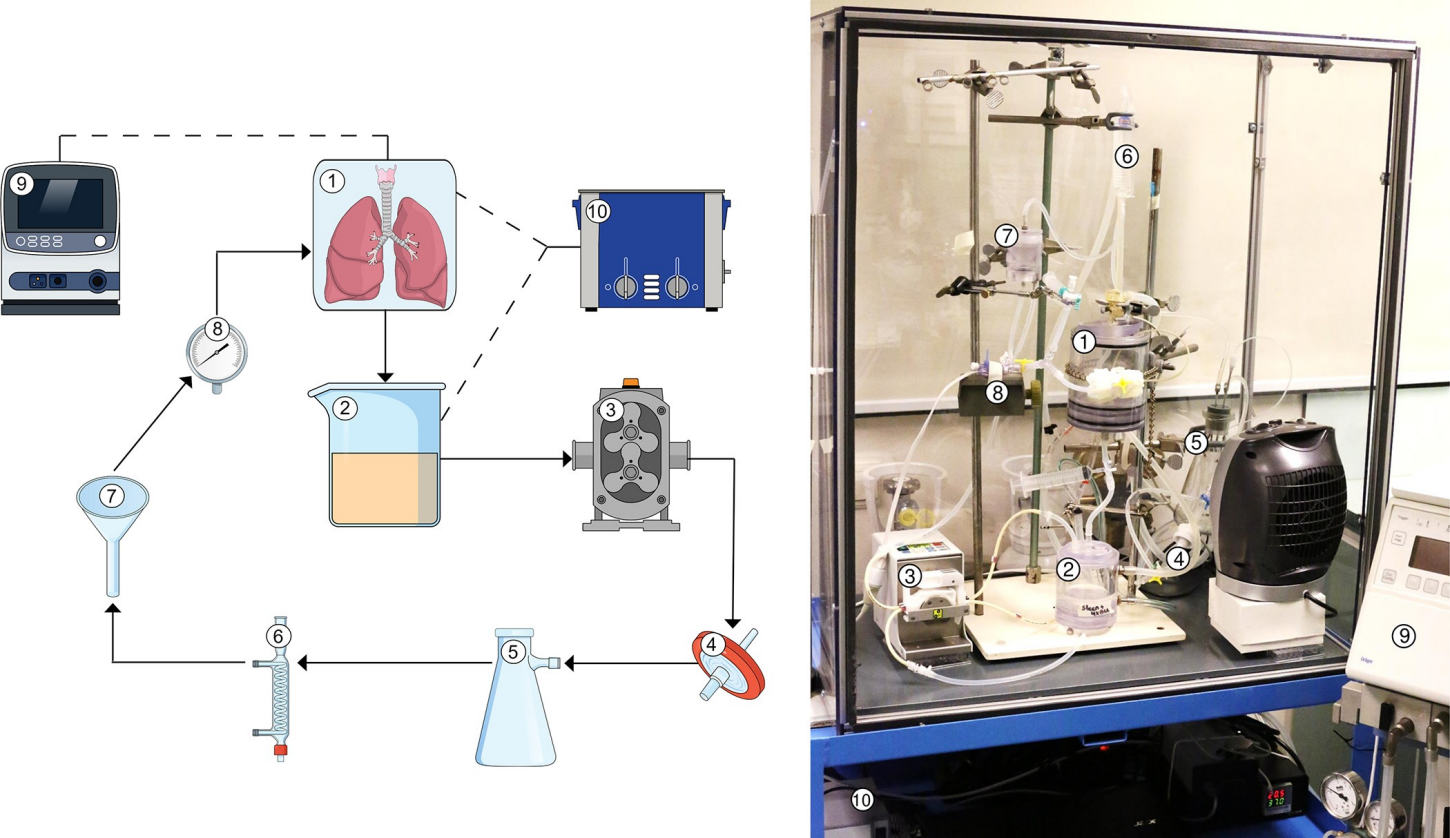
Schematic overview of the rat *ex vivo* lung perfusion model. Lungs from healthy or brain-dead donor rats were subjected to 6 hours *ex vivo* lung perfusion (EVLP). The EVLP circuit consisted of a (1) graft humidity chamber, (2) reservoir, (3) roller pump, (4) leukocyte filter, (5) deoxygenator, (6) heat exchanger, (7) funnel, (8) pressure sensor, (9) ventilator, and a (10) water bath.

Reagents required for EVLP were mentioned in Table 1 and instruments are outlined in Table 2. The EVLP circuit consisted of a graft humidity chamber, reservoir, roller pump, leukocyte filter, deoxygenator, heat exchanger, funnel, and a pressure sensor. A ventilator and water bath were additionally connected to the circuit (Fig 2). The funnel was added to the system to convert the pulsatile pump flow to a continuous flow, and pressure of flow was regulated by adjusting the height of the funnel. All components of the system were connected by silicone tubing and bubble traps were incorporated. To prevent heat loss during perfusion of lungs, the EVLP system was placed in an isolation chamber.

**Table 1:** List of reagents for *ex vivo* lung perfusion.

Reagent	Manufacturer
Bovine serum albumin	Sigma-Aldrich, Zwijndrecht, the Netherlands
Cefuroxime	Sandox, Almere, the Netherlands
Gas mixture (6% O2, 8% CO2, 86% N2)	SOL s.p.a., Monza, Italy
Glucose solution	Baxter B.V., Utrecht, the Netherlands
Perfadex	XVIVO Perfusion, Gothenburg, Sweden
Steen solution	XVIVO Perfusion, Gothenburg, Sweden

**Table 2:** List of instruments for the *ex vivo* lung perfusion model.

Instrument	Manufacturer
Deoxygenator	Hand-made of 6 m 1706 Python silicone tubing (QEW Engineered Rubber B.V., Hoogezand, the Netherlands)
Funnel	Research Support Facility UMCG, Groningen, the Netherlands
Heat exchanger	Radnoti LLC, Covina, USA
Humidity chamber	Research Support Facility UMCG, Groningen, the Netherlands
Isolation chamber	Research Support Facility UMCG, Groningen, the Netherlands
Leukocyte filter	Pall Corporation, New York, USA
Pressure sensors	Edwards Lifesciences, Irvine, USA
Reservoir	Research Support Facility UMCG, Groningen, the Netherlands
Roller pump	ISMATEC, Wertheim, Germany
Silicone tubing	Masterflex L/S 16 3.1 mm, Thermo Fisher Scientific, Waltham, USA
Water bath	JULABO, Boven-Leeuwen, the Netherlands

The perfusate was prepared one day before the experiment, to ensure absence of microbubbles that possibly developed due to homogenization of contents. The EVLP system was primed with 150 ml Steen solution at room temperature, supplemented with 6 g bovine serum albumin and 0.12 g cefuroxime. After 1 hour of CS, the lungs were connected to the ventilator. The trachea was wrapped with wet gauze dressings and lungs were covered with plastic foil to prevent dehydration during perfusion. A recruitment maneuver was performed as described before and thereafter, pressure-regulated volume controlled ventilation was initiated at the following settings: VT of 4 ml/kg of BW, PEEP of 5 cmH_2_O, I:E of 1:1 and FiO_2_ of 0.21. Subsequently, the pulmonary artery cannula was connected to the perfusion system and perfusion flow was initiated at a pressure of 9 mmHg. The water bath was started to gradually increase temperature to 37°C. After 10 minutes of reperfusion, VT was increased to 7 ml/kg of BW and perfusion pressure was adjusted to 12 mmHg. The first 5 ml of erythrocyte-rich perfusate exiting the lung were collected and discarded. Glucose levels of the perfusate were measured every hour and corrected with glucose solution (50 g/l) in case levels dropped <9 mmol/l. After 6 hours of EVLP, lungs were partially snap frozen and partially formalin-fixed, paraffin embedded for further analyses.

### Criteria for successful brain death and ex-vivo lung perfusion

The BD and subsequent EVLP procedure were considered successful if the following criteria were achieved: 1) successful inflation of the balloon catheter and confirmation of BD by absence of corneal reflexes and toe pinch assessments, 2) stable 3 hour BD period in which MAP >80 mmHg was maintained under maximum volume administrations of 5 ml/hour saline and 2 ml/hour HAES, 3) successful lung procurement without lung damage and adequate flushing, 4) stable ventilation of the lung on the EVLP platform at maximum ventilation pressures of 45 cmH_2_O sustained for 6 hours [8], 5) ability to achieve a stable outflow of perfusate from the left ventricle sustained for 6 hours and 6) create a stable inflow through the pulmonary artery cannula with a maximum MAP of 15 mmHg, sustained for 6 hours.

### Ventilation parameters and perfusion flow

PIP required to ventilate with VT of 7 ml/kg of BW was noted over time. Dynamic compliance (C_dyn_) was calculated by the following equation: C_dyn_ = VT/(PIP-PEEP). PaO_2_ was measured by an ABL 90 blood gas analyzer. Before sample taking, FiO_2_ was increased to 1 and the perfusate was deoxygenated for 5 min with a gas mixture of 6% O_2_, 8% CO_2_ and 86% N_2_. Oxygenation status was calculated by the PaO_2_/FiO_2_ ratio. Perfusion flow was determined by measuring the number of milliliters of perfusate exiting the left ventricle over 1 min.

### Metabolic profile analyses

Glucose and lactate levels pre- and post-lung were measured by means of blood gas analyses. Glucose consumption was calculated by the following equation: ΔGlucose = glucoseinflow−glucose_outflow_. Lactate production was calculated by the following equation: ΔLactate = lactateoutflow−lactate_inflow_. Glucose consumption and lactate production are presented as cumulative levels over time.

### RT-qPCR

Pro-inflammatory gene expressions were assessed by means of RT-qPCR. TRIzol reagent (Invitrogen Life Technologies, Breda, the Netherlands) was used according to manufacturer’s instructions, to extract total RNA from frozen lung tissue. RNA integrity was analyzed by gel electrophoresis and genomic DNA was removed with DNAse I (Invitrogen Life Technologies, Breda, the Netherlands). RNA to cDNA transcription was performed according to manufacturer’s instructions. RT-qPCR products were amplified by the Taqman Applied Biosystems 7900 HT RT-qPCR system (Applied Biosystems, Carlsbad, USA) and detected by measurement of SYBR Green emission (Applied Biosystems, Carlsbad, USA). Melt curve analyses confirmed generation of single, specific amplicons. Samples were measured in triplicate and gene expressions were normalized relative to house-keeping genes Ppia and Eif2b1. The ΔΔCt method was applied for calculation of gene expression levels [9].

### Quantitative lung edema measurements

Severity of lung edema was measured by wet/dry (W/D) ratio calculations of the right middle lobe, before and after drying for 24 hours at 100°C. W/D ratio was calculated according to the following equation: W/D ratio = (weight pre-drying–weight Eppendorf tube) / (weight post-drying–weight Eppendorf tube).

### Histological lung morphology

To assess histological lung morphology, formalin-fixed and paraffin embedded lung sections (4μm) were stained for hematoxylin and eosin (H&E). Per lung section, 10 random fields were quantified in a blinded manner on 400x magnification, based on a previously described lung injury score [10]. Five independent variables were scored: 1) inflammatory cell influx in interstitium and alveolar space, 2) alveolar septal thickening, 3) intra- and extra-alveolar hemorrhage, 4) intra-alveolar edema and 5) over-inflation. Variables were scored from 0–4: 0 = negative, 1 = slight, 2 = moderate, 3 = high and 4 = severe. The sum of the scored variables generated the total lung injury scores.

### Statistical analysis

Statistical analyses were performed with IBM SPSS Statistics 26 (IBM Corporation, New York, USA). Multiple comparisons between groups were analyzed by Kruskal-Wallis tests. In case of statistical significance, Mann-Whitney U post-hoc tests were performed to compare differences between two groups. Dependent variables measured over time were analyzed by mixed-model analyses of variance (ANOVA) tests, to analyze the effect of group and time. As follow-up tests, one-way ANOVA tests were performed with post-hoc Bonferroni tests to investigate differences between groups at specific time points. Statistical tests were 2-tailed and p<0.05 was considered statistically significant. Data are presented as mean ± standard deviations (SD).

## Results

**Fig 3:**
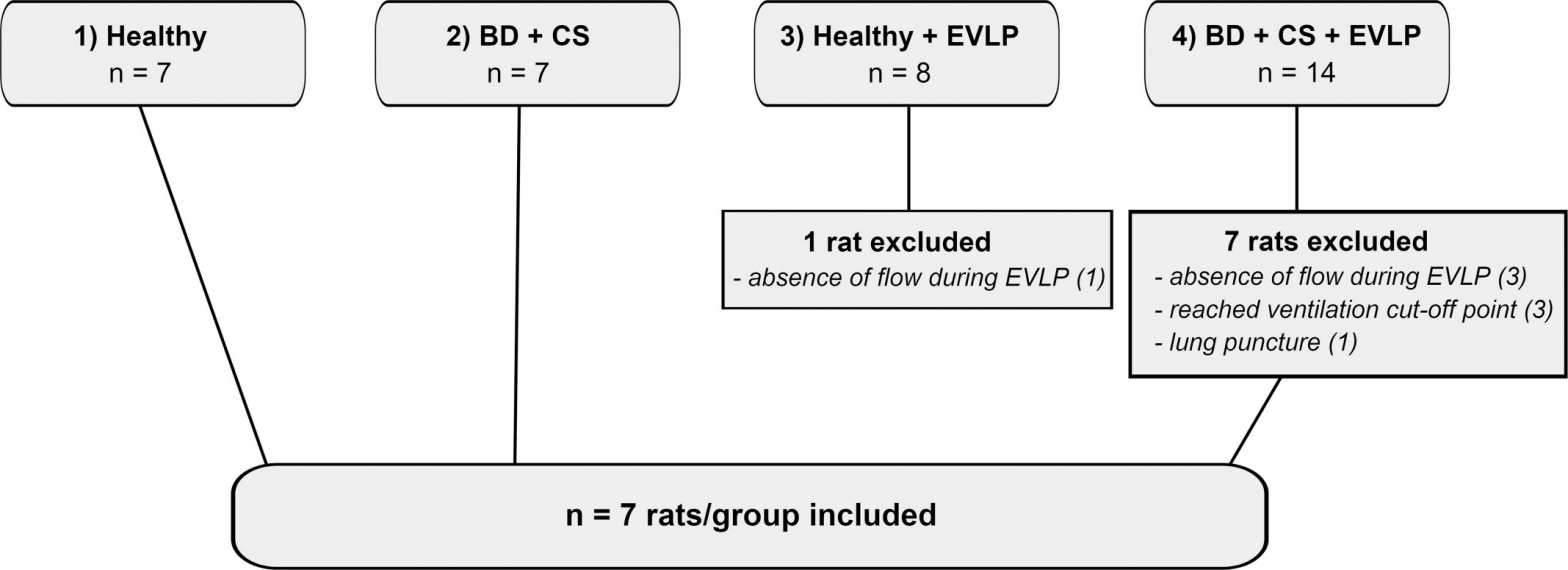
Experimental groups with number of rats utilized and rationale for exclusion. Lungs from donor rats were randomly assigned to one of four experimental groups (n = 7/group): 1) healthy, directly procured lungs, 2) lungs procured from rats subjected to 3 hours of brain death (BD) and 1 hour cold storage (CS), 3) healthy, directly procured lungs subjected to 6 hours *ex vivo* lung perfusion (EVLP) and 4), lungs procured from rats subjected to 3 hours of BD, 1 hour CS and 6 hours EVLP. In total, 38 rats were utilized for the establishment of a stable BD and EVLP protocol, of which 10 rats were excluded. Eventually, 7 rats per group were included in the final protocol.

In total 36 rats were subjected to either BD or a direct lung procurement, with or without EVLP (Fig 3). No rats were excluded from group 1, the direct procurement of healthy lungs, and group 2, the BD + CS group. In group 3, healthy donor lungs subjected to EVLP, 1 rat was excluded because of absence of perfusion flow during EVLP with unknown cause. In group 4, in which lungs from brain-dead donors were subjected to CS and EVLP, 7 rats were excluded. Of these, 3 rats were excluded because of absence of perfusion flow during EVLP, of which in 2 cases an air embolus was observed. Another 3 rats were excluded from group 4 because the ventilation cut-off point of 40 cmH2O PIP needed to maintain ventilation at 7 ml/kg of BW was reached, and 1 rat was excluded because of an observed puncture in the lung. Eventually, 7 rats per group were included in the final protocol, of which the results will be presented in further detail in this article.

### Brain death induction and blood pressure management

Acute traumatic BD was induced in rats assigned to experimental group 2 and 4, with or without EVLP. Upon induction of acute traumatic BD, a characteristic pattern of MAP was observed [1]. Baseline MAP was 148.86 ± 20.72 mmHg and after 3 hours of donor stabilization, MAP was 90.93 ± 14.68 mmHg. The mean total volume of saline and HAES administered to maintain MAP >80 mmHg was 12.4 ± 1.67 ml.

### Ventilation parameters, inflammatory status and lung morphology after lung procurement

**Fig 4:**
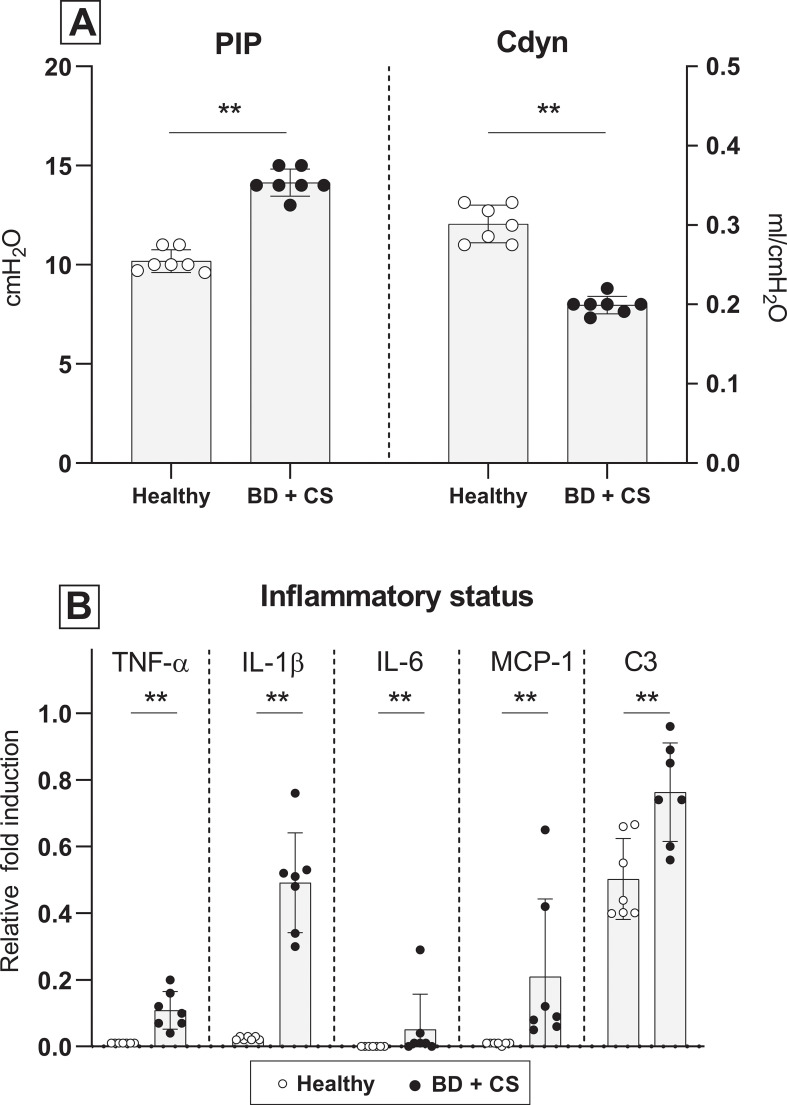
Ventilation parameters and inflammatory status after lung procurement. Lungs were procured from either healthy rats (experimental group 1) or rats subjected to 3 hours of brain death (BD, experimental group 2). (A) Pulmonary Inspiratory Pressure (PIP) required to maintain tidal volume at 7 ml/kg of bodyweight and dynamic compliance (C_dyn_) of healthy donor lungs 1) *versus* donor lungs subjected to 3 hours of BD, at time of lung procurement. (B) Pro-inflammatory gene expressions of TNF-α, IL-1β, IL-6, MCP-1 and C3 of healthy donor lungs *versus* donor lungs subjected to 3 hours of BD. ** p<0.01 in healthy donor lungs *versus* lungs from brain-dead donors.

**Fig 5:**
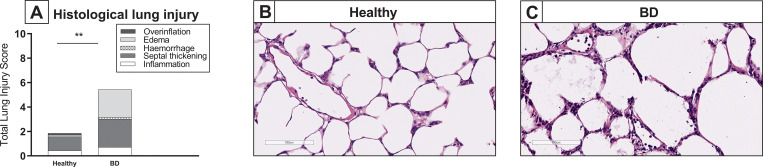
Lung morphology after lung procurement. Lungs were procured from either healthy rats (experimental group 1) or rats subjected to 3 hours of brain death (BD, experimental group 2). Histological lung injury was scored after staining for hematoxylin and eosin (H&E). (A) Quantification of lung injury scores in H&E-stained lung slides. (B-C) Representative H&E-stained slices of healthy donor lungs and lungs from brain-dead donors. ** p<0.01 in healthy donor lungs *versus* lungs from brain-dead donors.

To confirm the presence of lung damage and inflammation due to the pathophysiology of BD, we compared ventilation parameters, inflammatory status and lung morphology of healthy donor lungs to lungs procured from brain-dead donors [1]. PIP required to maintain VT at 7 ml/kg of BW was measured at the time of lung procurement, and was significantly higher in brain-dead rats when compared to healthy donor rats. In addition, C_dyn_ of rat lungs from brain-dead donors was significantly lower than C_dyn_ of healthy donor lungs at time of lung procurement (Fig 4A). Pro-inflammatory status of the donor lung was investigated by gene expression analyses of pro-inflammatory cytokines and histological influx of inflammatory cells. In lungs from brain-dead donors, pro-inflammatory gene expressions of TNF-α, IL-1β, IL-6, MCP-1 and central complement component C3 were significantly upregulated, compared to healthy donor lungs (Fig 4B). On a histological level, total lung injury scores were higher in lungs from brain-dead donors than in healthy donor lungs (Fig 5A–5C), which was mainly the result of a significantly increased influx of inflammatory cells. Presence of edema on a histological level seemed more evident in lungs from brain-dead donors, though not significant (p = 0.073). In addition, quantification of edema as reflected by W/D ratio, was comparable between groups (5.70 ± 0.18 in lungs subjected to BD *versus* 5.74 ± 0.42 in healthy donor lungs). These results indicate that our experimental BD model induced lung damage and inflammation, in line with the BD-induced pathophysiology as described in literature [11].

### Ventilation and perfusion performance during ex vivo lung perfusion

**Fig 6:**
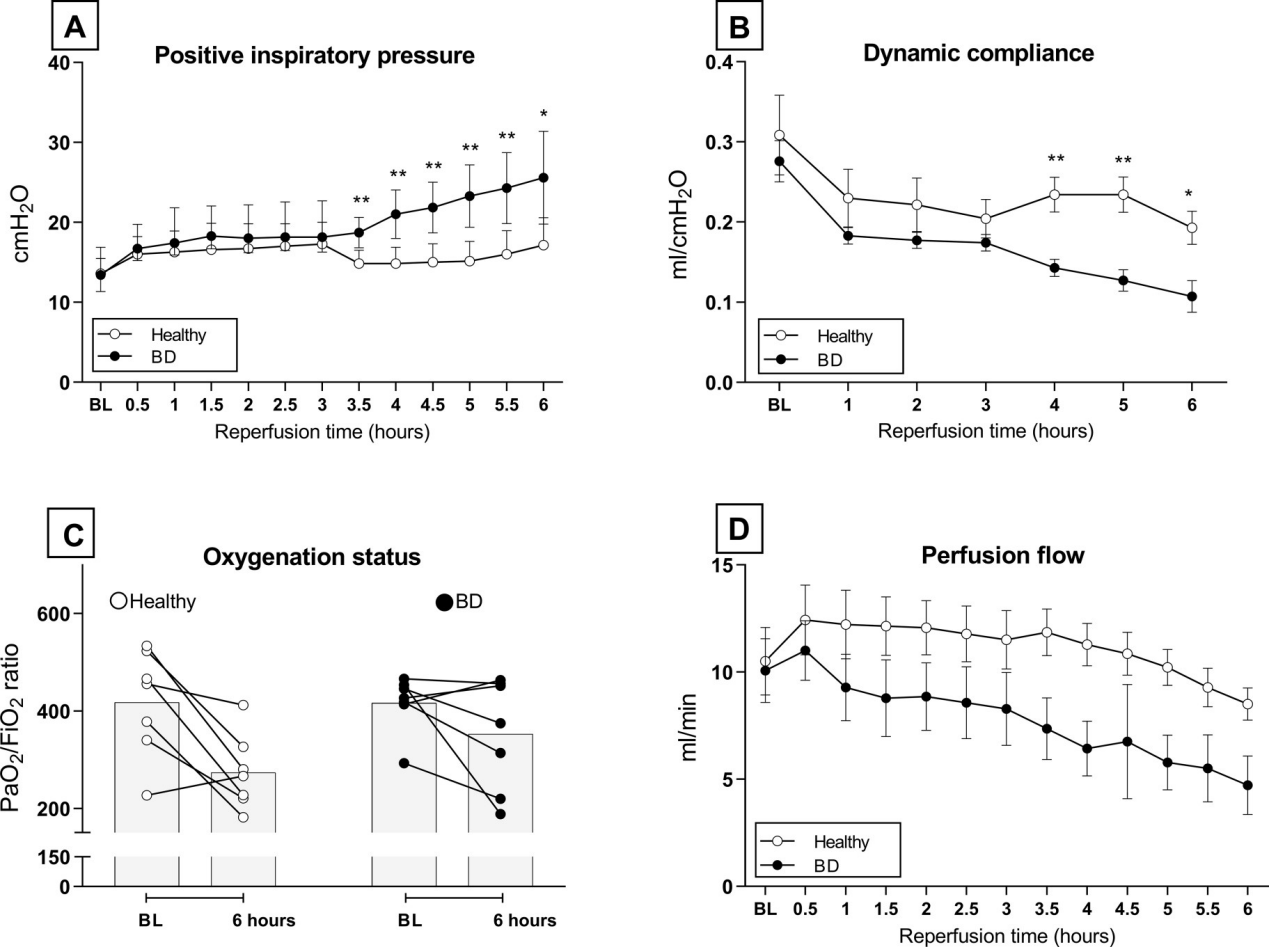
Ventilation and perfusion performance during *ex vivo* lung perfusion. Lungs from healthy donor rats or rats subjected to 3 hours of brain death (BD) and 1 hour cold storage (CS) were *ex vivo* perfused for 6 hours (EVLP, experimental group 3 and 4). (A) Positive Inspiratory Pressure (PIP) required to maintain tidal volume at 7 ml/kg of bodyweight over time, during EVLP. (B) Dynamic compliance (C_dyn_) of donor lungs over time, during EVLP. (C) Oxygenation capacity of donor lungs as reflected by PaO_2_/FiO_2_ ratio. (D) Perfusion flow of donor lungs over time, during EVLP. * p<0.05 in healthy donor lungs *versus* lungs from brain-dead donors subjected to EVLP. ** p<0.01 in healthy donor lungs *versus* lungs from brain-dead donors subjected to EVLP.

Lungs from healthy *versus* brain-dead donors were subjected to EVLP and in both groups, 7 lungs met the inclusion criteria for stable EVLP. Ventilation and perfusion parameters were compared between groups to investigate performance during EVLP. PIP required to maintain VT at 7 ml/kg of BW significantly increased over time in lungs from brain-dead donors, while lungs from healthy donors were stable over time. From 3.5 hours onward, lungs from brain-dead donors required significantly higher PIP than lungs from healthy donors (Fig 6A). C_dyn_ values showed a comparable pattern. Lungs from brain-dead donors showed a significant decrease in C_dyn_ over time, while C_dyn_ in lungs from healthy donors was stable over time. From 4 hours of reperfusion, C_dyn_ was significantly worse in lungs from brain-dead donors when compared to lungs from healthy donors (Fig 6B). Nevertheless, oxygenation capacity as reflected by PaO_2_/FiO_2_ ratio was not significantly different between groups (Fig 6C). With regard to perfusion, flow was significantly affected by time and was lower in lungs from brain-dead donors than in healthy donor lungs, though significance was not reached (Fig 6D). Collectively, these results show that a stable model for EVLP was established, in which lungs from brain-dead donors show worse ventilation performance than lungs from healthy donors.

### Metabolic profile, inflammatory status and lung morphology after ex vivo lung perfusion

**Fig 7:**
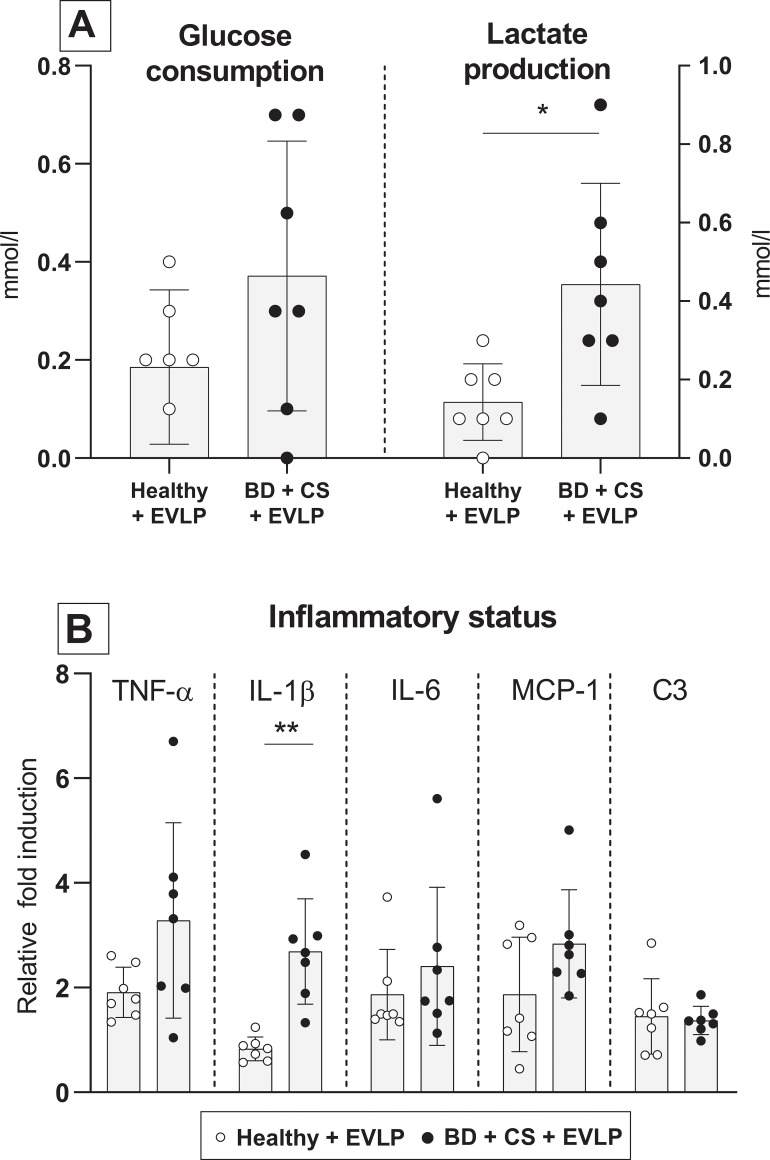
Metabolic profile and inflammatory status after *ex vivo* lung perfusion. Lungs from healthy donor rats or rats subjected to 3 hours of brain death (BD) and 1 hour cold storage (CS) were *ex vivo* perfused for 6 hours (EVLP, experimental group 3 and 4). (A) Cumulative glucose consumption of healthy donor lungs *versus* lungs from brain-dead rats, during EVLP. (B) Cumulative lactate production of healthy donor lungs *versus* lungs from brain-dead rats, during EVLP. (C) Pro-inflammatory gene expressions of TNF-α, IL-1β, IL-6, MCP-1 and C3 in donor lungs, after 6 hours of EVLP. * p<0.05 in healthy donor lungs *versus* lungs from brain-dead donors subjected to EVLP. ** p<0.01 in healthy donor lungs *versus* lungs from brain-dead donors subjected to EVLP.

Glucose consumption and lactate production were measured to investigate the metabolic profile of lungs during EVLP. While cumulative glucose consumption by lungs from brain-dead donors seemed higher than healthy donor lungs, no significance was reached (p = 0.196). Nevertheless, cumulative lactate production was significantly higher by lungs from brain-dead donors than by healthy donor lungs (Fig 7A). These results suggest that lungs from brain-dead donors shift to an anaerobic metabolism, which does not occur in lungs from healthy donors. Pro-inflammatory status of donor lungs after EVLP was investigated by gene expression analyses of pro-inflammatory cytokines and histological influx of inflammatory cells. Overall, pro-inflammatory gene expressions were elevated in lungs from brain-dead donors compared to healthy donor lungs. However, only IL-1β gene expression reached significance (p = 0.002), in contrast to TNF-α (p = 0.085), IL-6 (p = 0.224) and MCP-1 (p = 0.277). Gene expressions of C3 were similar between healthy donor lungs and lungs from brain-dead donors (p = 0.749, Fig 7B).

**Fig 8:**
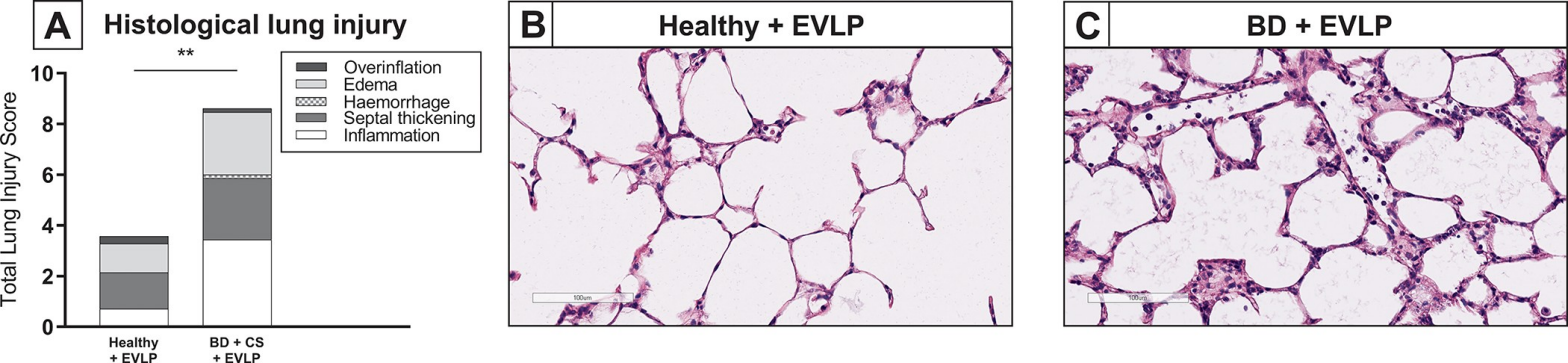
Lung morphology after *ex vivo* lung perfusion. Lungs from healthy donor rats or rats subjected to 3 hours of brain death (BD) and 1 hour cold storage (CS) were *ex vivo* perfused for 6 hours (EVLP, experimental group 3 and 4). Histological lung injury was scored after staining for hematoxylin and eosin (H&E). (A) Quantification of lung injury scores after EVLP in H&E-stained lung slides. (B-C) Representative H&E-stained slices of healthy donor lungs and lungs from brain-dead donors, after EVLP. ** p<0.01 in healthy donor lungs *versus* lungs from brain-dead donors subjected to EVLP.

On a histological level, total lung injury scores after EVLP were higher in lungs from brain-dead donors than in healthy donor lungs (Fig 8A–8C), which was mainly the result of an increased amount of inflammatory cells. Presence of edema on a histological level was comparable between lungs from brain-dead donors and healthy donor lungs subjected to EVLP (p = 0.429). In accordance, the amount of lung edema as reflected by W/D ratio, was similar between healthy lungs and lungs from brain-dead donors (respectively 6.10 ± 0.31 *versus* 6.24 ± 0.43). Taken together, these results suggest that both healthy donor lungs and lungs from brain-dead donors show an increase in inflammatory status after reperfusion, which seems more evident in pre-damaged donor lungs from brain-dead donors.

## Discussion

The technique of EVLP rapidly emerges in the clinical field of lung transplantation. While originally designed to test and assess marginal donor lungs, the potential of EVLP as a treatment platform is now increasingly being investigated [12]. Rat models for EVLP provide a reproducible, cost- and time effective manner to perform experimental studies, due to small animal size [13]. However, rat EVLP models currently described in literature, are commonly performed with lungs procured from living, anesthetized rats or from rats deceased after circulatory arrest, and require anti-inflammatory additives to enable perfusion for more than 3 hours (Table 3) [14]. Since clinically most donor lungs are procured from brain-dead donors, we aimed to set up a stable model for rat EVLP with pre-injured lungs from brain-dead donors to increase translatability to the clinical setting. This report details the technical aspects of stable, prolonged EVLP of lungs from brain-dead rats without addition of anti-inflammatory agents, which can be applied in future studies focused on BD-induced lung injury and potential treatment modalities.

**Table 3:** Donor type characteristics of reported rat *ex vivo* lung perfusion models.

Author, ref	Donor type	Mode of euthanasia
Bassani [15]	Living donation	Exsanguination
Dacho [16]	Living donation	Exsanguination
Davis [17]	Living donation	Exsanguination
Francioli [18]	DCD	Exsanguination, lung left in situ for 1 hour warm ischemia
Hirata [19]	Living donation	Exsanguination
Hijiya [20]	DCD	Withdrawal of hemodynamic and ventilatory support
Hodyc [21]	DCD	Overdose sodium thiopental
Inokawa [22]	DCD	Overdose intrahepatic pentobarbital sodium
Liu [23]	Living donation	Exsanguination
Markou [24]	Living donation	Exsanguination
Motoyama [25]	DCD	Airway occlusion
Nelson [26]	Living donation	Exsanguination
Noda [14]	Living donation	Exsanguination
Ohsumi [27]	Living donation	Exsanguination
Pêgo-Fernandes [28]	Living donation	Exsanguination
Roffia [29]	Living donation	Exsanguination

The BD protocol used in this report is modified from previously described BD models by our group [6, 7]. The mentioned models were developed with emphasis on BD-induced liver and kidney injury and describe both fast and slow induction models, which each represent different clinical pathophysiological mechanisms of BD. Traumatic brain injury reflects a fast mode of BD, while cerebrovascular events such as hemorrhagic stroke usually refer to a slower induction of BD [6, 30]. In this protocol, we preferred fast BD induction over slow BD induction, since previous findings by our group show that fast BD leads to more lung damage than slow BD induction, which provides an additional challenge to establish a stable protocol when combined with EVLP [31].

The clinically described pathophysiological features associated with donor BD are mimicked in our rat BD model, which therefore provides a reliable setting for experimental BD studies. The process of BD resulted in lung injury and inflammation in our model, corroborated by worsened ventilation parameters, upregulated pro-inflammatory gene expression levels and increased histological injury scores in lungs from brain-dead donors compared to healthy donor lungs [11, 32, 33]. The deteriorated quality of lungs from brain-dead donors compared to healthy donor lungs was evident after reperfusion on the EVLP platform as well. Ventilation performance of lungs from brain-dead donors worsened during EVLP as corroborated by PIP and C_dyn_ values, while healthy donor lungs showed a stable course over time. Nevertheless, oxygenation status was not affected, although the reliability of this test is questioned in the condition of acellular perfusate and an open perfusion system. In plasma-like solutions only few oxygen molecules can significantly change PaO_2_ values, which suggests that lung compliance may be a more accurate parameter to assess lung quality [34]. Metabolically, the process of BD is described to induce a change from aerobic to anaerobic metabolism [35]. In our study, this anaerobic shift was indeed evident in lungs from brain-dead donors as corroborated by increased lactate production during EVLP, when compared to healthy donor lungs. Ischemia/reperfusion injury (IRI) was evident in lungs from brain-dead donors as well as in healthy donor lungs, as corroborated by generally higher pro-inflammatory gene expressions after 6 hours of EVLP compared to the inflammatory state at the time of lung procurement. The overall trend showed a more evident pro-inflammatory augmentation in lungs from brain-dead donors compared to healthy donor lungs, yet significance was only reached for IL-1β gene expressions. These observations possibly reflect the ‘double hit’ damage model of the transplantation process, in which BD exacerbates IRI [36].

In addition to the ‘double hit’ damage model, other factors were carefully considered in this protocol to increase translatability to the clinical setting. Bassani *et al*. previously provided an overview of perfusion lengths of various rat EVLP models described in literature, and emphasized the importance of prolonged perfusion length to reproduce the clinical condition. While most experimental rat EVLP models did not achieve perfusion for longer than 30–120 min, Bassani *et al*. established a stable rat EVLP model for 3 hours [15]. Clinical protocols generally recommend to perfuse for a minimum of 3 hours in the evaluation of lung function, before a final decision can be made [37]. For future *ex vivo* repair strategies or prognostic testing, even longer perfusion lengths are pursued [38]. Our rat EVLP model met the criteria for stable EVLP up to 6 hours of perfusion, which we consider an adequate basis for experimental studies.

The establishment of a prolonged perfusion length in small animal models is challenging, because of their higher susceptibility to lung edema and atelectasis compared to larger animal models [13]. One of the key aspects that enabled a perfusion length of 3 hours in the rat EVLP model described by Bassani *et al*. was the step-wise initial reperfusion phase, which included a gradual increase of flow rate, tidal volume and temperature [15]. In most described rat EVLP models flow rate is a set parameter, calculated based on the estimated cardiac output. The corresponding pulmonary arterial pressures seem variable, albeit not registered for every described model [15]. Low pulmonary arterial perfusion pressures is a key feature for successful EVLP [38, 39]. To minimize lung edema formation, we therefore chose to perfuse rat lungs with a non-pulsatile pressure-controlled strategy at 12 mmHg, after an initial perfusion pressure of 9 mmHg for 10 min. Corresponding flow rates ranged from 4.7–12.4 ml/min, which is in line with flow ranges described for previously established models [15]. In addition, we supplemented the perfusate with bovine serum albumin to increase colloid osmotic pressure, since lungs of small animals are more susceptible to edema compared to larger animal models due to their small organ size [13]. When we established the rat EVLP model, we observed that prolonged perfusion of rat lungs >4 hours was better achieved with supplementation of the perfusate with bovine serum albumin due to development of lung edema. Therefore, especially in the case of prolonged perfusion, we would suggest addition of bovine serum albumin to the Steen solution in this model. In addition, the effect of oncotic pressure may decrease over time, when oncotic pressure of perfusate and interstitial spaces equilibrate. In the clinical setting, Steen solution is replaced every hour of lung perfusion, which may be required to maintain stable levels of oncotic pressure in the circulating perfusate. However in our rat model we chose to not replace the perfusate during perfusion, to enable analysis of excretion components such as lactate and IL-6 over time. Nevertheless, the resulting osmolality of the perfusate at baseline was 289.33 ± 7.10 mOsm/kg, which, interestingly, is still within the range of osmolality of clinically used Steen solution [40]. We believe that further dose-dependent studies are needed to determine the exact concentration of additional bovine serum albumin required to prevent lung edema formation in prolonged perfusion of rat lungs.

An additional key aspect for prolonged perfusion is a lung-protective ventilation strategy, which we initiated at tidal volumes of 4 ml/kg and increased to 7 ml/kg after 10 min. Perfusate temperatures were increased from room temperature to physiological values in the initial reperfusion phase, although no strategized slow-rewarm approach was included in our model. Another strategy applied by previously described models to prolong perfusion length is the addition of anti-inflammatory agents to the perfusate [14, 21]. Although successful in prevention of edema formation, a stable rat EVLP model without anti-inflammatory agents is preferred to enable experimental studies focused on immunomodulatory mechanisms. With the before mentioned reperfusion strategy, we achieved stable EVLP for 6 hours without addition of anti-inflammatory agents.

Trial and error developments of the BD and EVLP procedure allowed us to identify key learning points, which we outline here to facilitate reproduction by other research groups. As for the BD procedure, excessive blood loss should be avoided to minimize MAP instability during the BD period. In addition, the Fogarty catheter for induction of BD should be carefully inspected for air bubbles and must be left inflated until termination of the experiment. We recommend to stabilize the brain-dead donor rat on a MAP >80 mmHg with saline and HAES administrations, though at a maximum of 5 ml/h saline and 2 ml/h HAES to prevent edema formation. If MAP does not respond to these measures or volume limitations are reached, venous backflow to the heart can be increased by elevation of the legs. Hypothermia should be avoided to prevent dysfunction of the heart and coagulation cascades, for which we recommend to use a surgical heating pad and, if required, an additional heating lamp [41]. At time of lung procurement, lung damage should be avoided and we therefore advise to carefully inspect the donor lung before proceeding with the protocol. In order to reduce the risk of lung damage by sharp rib ends, we decreased PEEP to 3 cm H_2_O and respiratory rate to 60/min to enhance visibility during the lung procurement procedure. Lungs were flushed with Perfadex at room temperature, which was previously described by our group to be more beneficial for lung graft preservation than cold flush [42]. The flush cannula should be carefully inspected for air bubbles, since introduction of air during lung flush leads to tissue damage and increased pulmonary vascular resistance. No retrograde flush was performed in contrast to clinical protocols, since we did not cannulate the left atrium. By cannulation of the left atrium a physiological slight positive atrial pressure can be maintained, which is known to protect the pulmonary vasculature. However, we chose for an open-atrium strategy by incising the left ventricle with destruction of the mitral valve. We preferred the open-atrium strategy in our model, since a downside of left atrial cannulation is possible unintended high left atrium pressures or negative pressures during EVLP, which cause lung injury and air bubble formation in the EVLP system [43].

During EVLP air bubbles should be avoided, therefore we recommend inclusion of bubble traps in the system and careful priming of the perfusion system. The occurrence of an air embolism during EVLP might become evident by a sudden decrease of flow for a given perfusion pressure. Although, the same observation might result from a dislocated pulmonary artery cannula. Similarly, a twisted tracheal cannula might cause a sudden inability to reach aspired tidal volumes. Therefore, we advise to inspect and confirm the correct position of both ventilation and perfusion cannulas regularly.

This report describes for the first time a rat EVLP model with pre-injured donor lungs from brain-dead donors. Small rodent models share advantages over large animal models in terms of cost effectiveness and reproducibility, which makes our current described rat model an excellent starting point for experimental studies. Nevertheless, large animal models might be preferred in follow-up studies, given their comparable size to human donor lungs and thereby closer translatability to the clinics [13].

In our current rat EVLP model, we chose to compare outcomes of lungs from brain-dead donor rats to outcomes of lungs from healthy donor rats. As a consequence, lungs of the healthy control group were not subjected to 3 hours ventilation and 1 hour CS, which is a limitation of our study. Nevertheless for well-considered reasons, a healthy control group was preferred. First, the described anti-inflammatory effects of sedatives such as volatile anesthetics or ketamine, may interfere with our established inflammatory end-points when applied for 3 hours in sham-operated rats [44, 45]. In addition, we previously experienced the challenging titration of the pursued depth of sedation in small rodents. Too little sedation may lead to breathing against the ventilator which damages the healthy donor lung, while too deep sedation may lead to hypotension and subsequent death of de donor rat. Last, we considered the use of a healthy control group a better comparison to the rat EVLP models described in literature so far, which are commonly performed with lungs procured from healthy donor rats.

In conclusion, we established a stable rat EVLP model for pre-injured lungs from brain-dead donors, which contributes to future studies on mechanisms of BD-induced injury, procedural adjustments and pharmacological interventions. We consider this report to be of importance to transplantation scientists, since a detailed protocol might facilitate reproduction by other research groups and reduce the required number of laboratory rats, in favor of the 3R principles of animal research.

## Supporting information

S1 Dataset Dataset of Figs 4–8. (XLSX)
